# Novel Optical Sensor-Based Organic Chromophore for the Detection of Copper Ions

**DOI:** 10.3390/bios16080436

**Published:** 2026-08-12

**Authors:** Reham Ali, Majd K. Almotiri, Sabri Messaoudi, Ibrahim A. I. Ali, Azizah Algreiby, Sayed M. Saleh

**Affiliations:** 1 Department of Chemistry, College of Science, Qassim University, Buraidah 51452, Saudi Arabia; re.ali@qu.edu.sa (R.A.); majd.k.almotiri@gmail.com (M.K.A.); s.messaoudi@qu.edu.sa (S.M.); 2 Chemistry Department, Faculty of Science, Suez Canal University, Ismailia 8366004, Egypt; ibrahim_ali@science.suez.edu.eg

**Keywords:** chemosensor, fluorescence probe, environmental monitoring, limit of detection, quinoline derivatives, copper

## Abstract

During this research, a distinctive optical sensor film was devised and designed to detect Cu(II). The 3-acetyl-4-hydroxyquinolin-2(1H)-one (*AHQ*) sensor probe is effectively synthesized. This organic probe of the sensor film exhibits exceptional sensitivity to Cu(II) ions and a “turn-off” state. This innovative fluorescent chemosensor is distinguished by its unique optical characteristics, which include a significant Stokes shift of approximately 91 nm. The binding of Cu(II) with *AHQ* organic probe introduces a 1:2 (metal:ligand) complex, accompanied by the quenching of the maximum emission peak at 455. Furthermore, *AHQ* exhibits exceptional selectivity for Cu(II). The quenching of the complex fluorescence is attributed to internal charge transfer (ICT), as indicated by the mechanism. The *AHQ* sensing molecule for Cu(II) ions is attributed to chelation-quenched fluorescence. Density functional theory (DFT) and time-dependent DFT (TDDFT) were employed to study the binding of Cu(II)–AHQ structures and related electronic characteristics in solutions. The results reveal that the luminescence quenching of this complex is caused by ICT. The influences of the interference ions were investigated using a solution that contained multiple metal ions. This *AHQ* molecule exhibits exceptional selectivity and sensitivity and a low LOD of 10.8 nM, and is administered in a physiological pH medium (pH = 7.4) with a relative standard deviation (RSD_r_) (1%, *n* = 3). Also, the *AHQ* shows good binding behaviour towards Cu(II), and the binding constant was determined to be 3.8 × 10^6^ M^−1^. As a result, these unique characteristics allow it to identify Cu(II) within a controlled dynamic range of 0.019–2.4 μM Cu(II). The reversibility of the chemosensor was established by using EDTA as a strong chelating agent. As a highlight, we present an important optical chemosensor dependent on the AHQ molecule.

## 1. Introduction

Copper has a critical influence on human health, as it is necessary for the development of bones and the respiration of cells, among other physiological processes [1,2]. Nevertheless, it is also highly toxic to living organisms in high concentrations, resulting in fatal neurodegenerative conditions such as Parkinson’s, Alzheimer’s, and hereditary aceruloplasminemia [3,4]. Furthermore, Cu(II) is a substantial environmental pollutant due to its widespread use in producing electronic devices, as well as in other industrial and agricultural operations. The World Health Organization (WHO) has established an acceptable level of copper in potable water at 2 ppm (31.5 μM) [5]. Consequently, there is a great demand for the design of chemosensors for the sensing and monitoring of Cu(II) with extreme sensitivity, significant LOD, and a fast response [6,7]. Cu(II) is also the third most prevalent metal ion required by all living creatures and is vital for many functions. On the other hand, the absence or excess of Cu(II) ions, depending on the needs of the cell, can lead to the formation of several illnesses. Optical chemosensors have been designed for the real-time detection of Cu(II) ions with high selectivity and sensitivity. Optical chemosensors operate by converting the presence of an analyte into measurable changes in optical properties, most commonly variations in absorption or fluorescence emissions and, in some platforms, shifts in the refractive index. This is because of the interaction of light and substance [8,9,10]. Recently, optical chemosensors have attracted great attention due to their simplicity, naked-eye identification, real-time recognition, low cost, remarkable specificity against analytes, fast reaction, and reduced equipment requirements throughout the research process [11,12]. Chemosensors are molecules or nanoparticles that alter one or more of their properties when they meet a foreign species [13,14]. These properties include colourimetric and fluorescent sensors for Cu(II) detection, which have attracted increased interest because of their affordability, fast reaction times, and suitability for in situ analysis [15,16,17].

The AHQ probe is the primary structural component of numerous bioactive natural compounds. The distinctive and diverse pharmacological properties of these classes of compounds—including their antimicrobial nature [18], their role as orally active antagonists [19], and their antitubercular [20], anticancer [21], and antimalarial [22] properties—are their fundamental structural characteristics [23,24]. In addition, research on these fascinating compounds has demonstrated that selective glycine site antagonists are associated with numerous neural disorders, such as Alzheimer’s disease, Parkinson’s disease, stroke, and death [25,26,27]. Quinolinone compounds have been widely employed as chemical probes and chemophores in chemosensors because of their exceptional optical stability and strong attendance towards metal ion reactions, as well as their unique structural characteristics and critical biological functions [28,29,30].

Trace metals can be detected using a variety of methods. High-performance liquid chromatography (HPLC) [31] and inductively coupled plasma mass spectrometry (ICP) [32] are frequently employed in copper ion sensing. However, these methods have drawbacks in terms of their reliability, precision, and rapidity, which should be accounted for. They are also costly and challenging to use [33,34]. Improving the sensitivity, simplicity, speed, repeatability, affordability, and analytical capabilities of these methods of tracing Cu(II) ions in the environment is imperative. The unique binding between the receptor and the specified analyte is the foundation of the detection mechanism. Moreover, the detection procedure may need to control various factors, such as pH, electrons, mass transfer, temperature, and potential alterations. This procedure converts the receptor’s response into a substantial signal that is precisely relative to the occurrence or amount of the metal ions [35,36]. In recent years, the prevalence and significance of sensor development have increased markedly due to the sensors’ rapid generation of semi-quantitative data, ease of miniaturization, and inherently low cost. Thus, optical sensors are invaluable tools in the detection of chemical and biological contaminants, helping us address important questions in environmental and nutritional research. Trace metals such as Cu(II), Hg(II), and Cd(II) are of increasing concern due to their persistence, bioaccumulation potential, and toxicity, which can lead to severe ecological impacts and adverse health effects through long-term exposure. Thus, the development of robust and sensitive optical chemosensors for the monitoring of these species in complex matrices is of paramount importance in modern analytical chemistry and environmental surveillance [37,38].

**Scheme 1:**
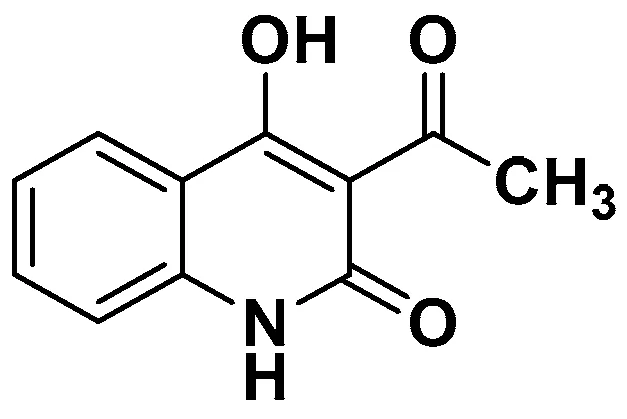
*AHQ* structure.

We have developed a novel chemosensor with unique optical characteristics to detect copper ions, including the quinolone groups, as shown in Scheme 1. The metal ion would be bound by the multiple-group chemosensors, modifying one or more of the system’s characteristics, including fluorescence and colour. This investigation delineates an organic probe sensor instrument that functions as an off switch. Our group has created a highly selective, reversible, and vulnerable sensor with a significant LOD and exceptional sensitivity for Cu^2+^ detection. Additionally, the Cu^2+^ detection procedure is predicated on the ICT mechanism. The developed and designed AHQ ligand is a distinctive chemosensor for tracing Cu^2+^. The determination method is predicated on the measurement of the luminescence quenching of the AHQ probe during the binding procedure, which depends on the Cu^2+^.

## 2. Materials and Methods

### 2.1. Chemicals and Apparatus

All reagents were acquired from Sigma-Aldrich and were utilized without any additional preparation. An EvolutionTM 200 series UV–visible spectrophotometer collected the UV-vis spectra. A JASCOFP6300 spectrofluorometer was used to the detect fluorescence spectra for each series of experiments. A stock solution of 20 mM HEBES buffer at a pH of 7.4 has been prepared. The ^1^H and ^13^C-NMR spectra were obtained using a Bruker NMR instrument that operates at 800 and 125 MHz. The chemical shifts were determined, with TMS serving as the standard internal reference and DMSO-d6 as the solvent. Using the Stuart SMP30 apparatus, the melting points were determined. A 254 nm UV lamp was used to obtain the chromatogram, and Silica Gel 60 F254 plates (40–60 m) from Sigma were utilized for analytical TLC. No further purification was performed on the glassware used; all experiments were conducted in nitrogen-protected dry air.

### 2.2. Procedure for AHQ Synthesis

A mixture of (1.5 mL, 12 mmol) of ethyl acetoacetate in 5 mL of xylene was agitated and heated under reflux as a solution of 1.5 mL (12 mmol) of methyl anthranilate in 4 mL of xylene was added drop by drop. The net solution was agitated and refluxed under reflux overnight and cooled to room temperature; then, 10 mL of 10% (*w*/*v*) aq. NaOH solution was inserted, and the mixture was stirred for 1 h. The resulting precipitate was filtered and washed with 20 mL of water, giving 1.07 g (45%) of *AHQ* as a white solid, mp. 252–254 [39,40].

### 2.3. Fabrication of Chemosensor Film

**Scheme 2:**
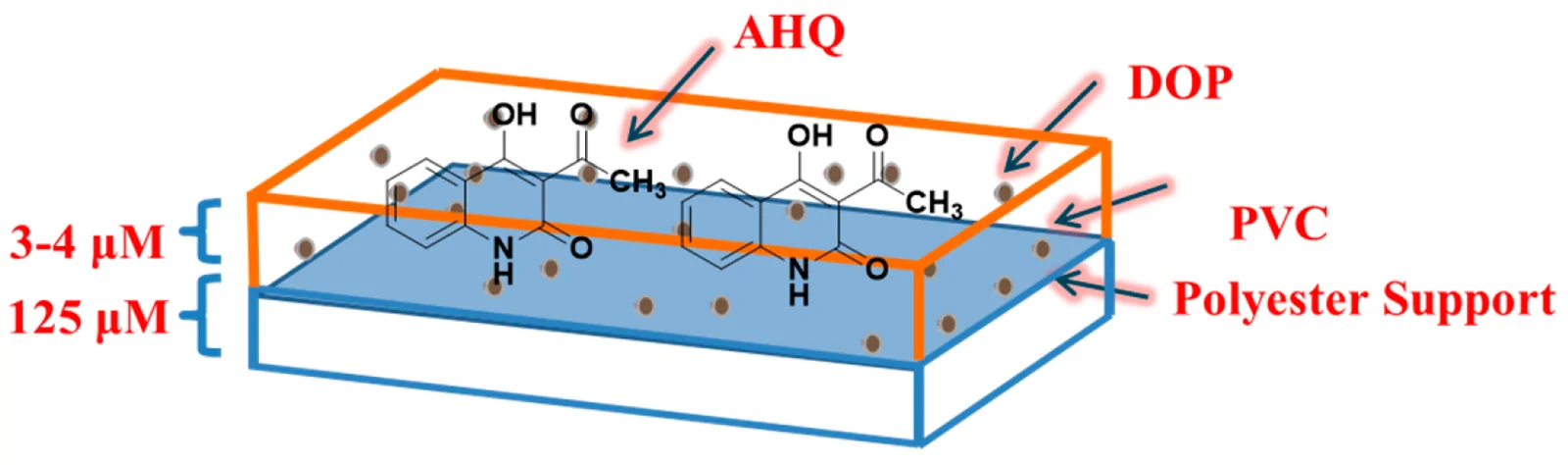
*AHQ* sensor film.

The optical sensor-film was produced by producing a cocktail of 2.25 mg of *AHQ*, 22.8 mg of PVC, poly(vinyl chloride), and 47.2 mg of DOP, dioctyl phthalate, in 2 mL of THF. In addition, the cocktail was agitated for approximately eight hours until it achieved a transparent solution. The concoction was applied to a polyester polymer support utilizing a knife coater [41]. The chemosensor was permitted to dry in the air. The thickness of the upper layer over the polymer support was approximated to be 3–4 μm depending on the content of the materials (see Scheme 2).

### 2.4. Optical Measurements

Various types of metal ions, all produced as nitrate ions, such as Al(III), Na(I), and others, were investigated, and each had its stock solution created. A luminescence titration was carried out with a native *AHQ* concentration of 2 μM in a buffer solution of 20 mM DMSO-HEPES containing (40/60, *v*/*v*) with a pH of 7.4 and 1 μM of Cu(II). Additionally, an aqueous solution with a concentration of 2 μM in 20 mM DMSO-HEPES containing (40/60, *v*/*v*) with pH of 7.4 was used to determine the absorbance maxima of both free *AHQ* and *AHQ* in the presence of 1 μM of Cu(II). The total volume of *AHQ* and M^n+^ ions can be considered to be 2.0 mL during absorption titration. This is because the M^n+^ volume can be ignored with respect to the *AHQ* volume. Throughout each experiment, excitation and emission slits were adjusted to be 5 nm.

## 3. Results

### 3.1. AHQ Synthesis

**Scheme 3:**
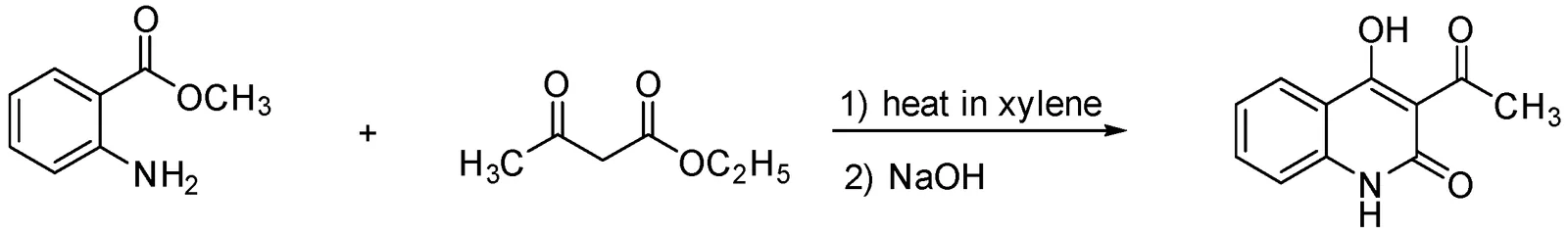
*AHQ* synthesis.

Scheme 3 presents the synthesis reaction of the chemical probe AHQ. The reaction yield was gathered by filtration and washed with water, giving 1.07 g (45%) of 3-acetyl-4-hydroxyquinolin-2(1H)-one as a white solid, mp 256–257 °C (lit., 78 252–254, 248–250 °C); ^1^H NMR (300.o MHz, DMSO-d6) δ 15.02 (bs, 1H, OH), 11.46 (bs, 1 H, NH), 7.95–7.06 (m, 4 H, Ar-H), 2.67 (s, 3 H, CH_3_); ^13^C NMR (75.0 MHz, DMSO-d6) δ 205.2 (CO), 170.6 (CO), 140.5, 134.9, 132.5, 114.4, 111.6, 110.4 (Ar-C), 29.7 (CH_3_); MS m/z 203.1. All data analyses of the synthesized AHQ are provided as shown in Figures S1–S3 in the Supporting Information.

### 3.2. AHQ Probe Properties

**Figure 1:**
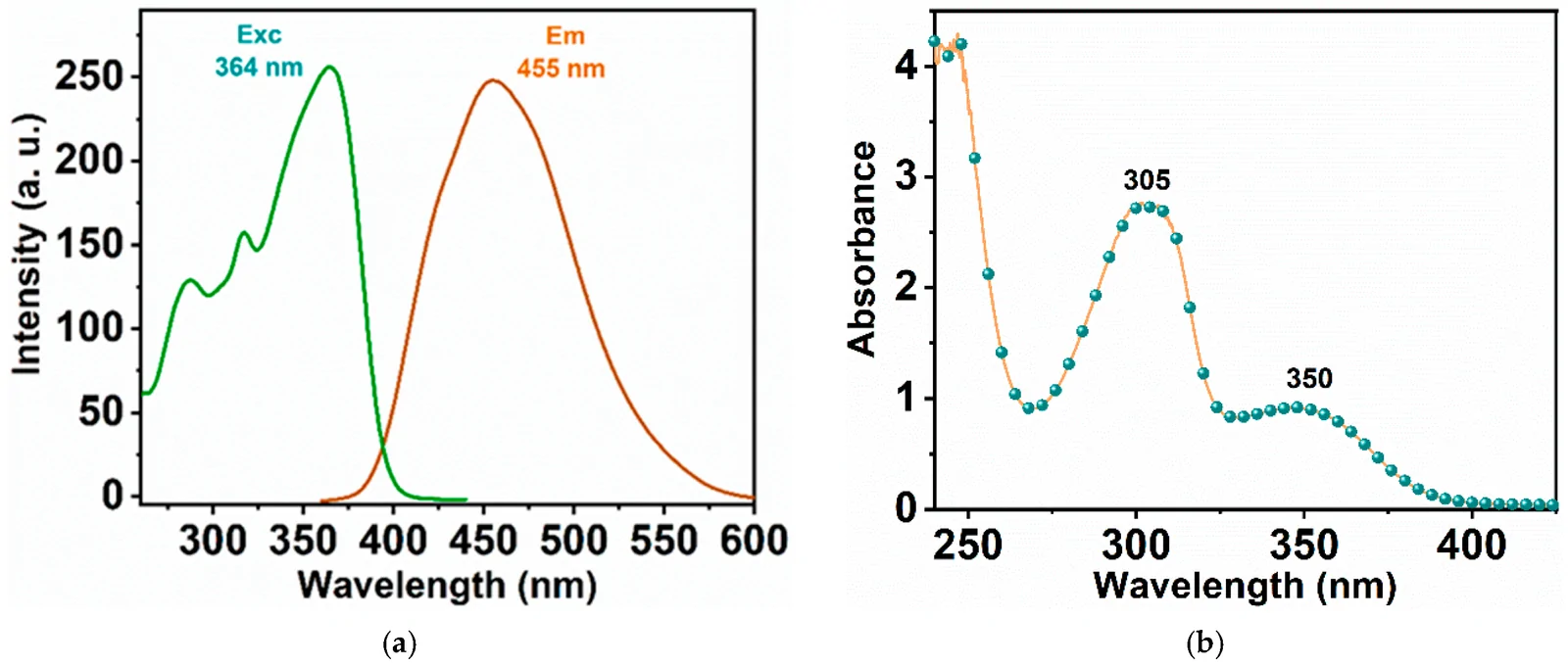
(**a**) Fluorescence response of 1 μM *AHQ* (red line) under 364 nm excitation (green line). (**b**) UV-vis spectrum of the *AHQ* as prepared ligand.

The *AHQ* chemical probe exhibits unique optical properties; these countenances were composed in a (40/60, *v*/*v*) DMSO-HEPES with a pH of 7.4 (see Figure 1). The *AHQ* chemical probe under excitation at 364 nm produces a well-defined luminescence peak at 455 nm, as shown in Figure 1a. Also, the UV-vis absorption spectrum provides two maximums at 305 and 350 nm, respectively (see Figure 1b).

### 3.3. pH Influence on the AHQ Emissions

**Figure 2:**
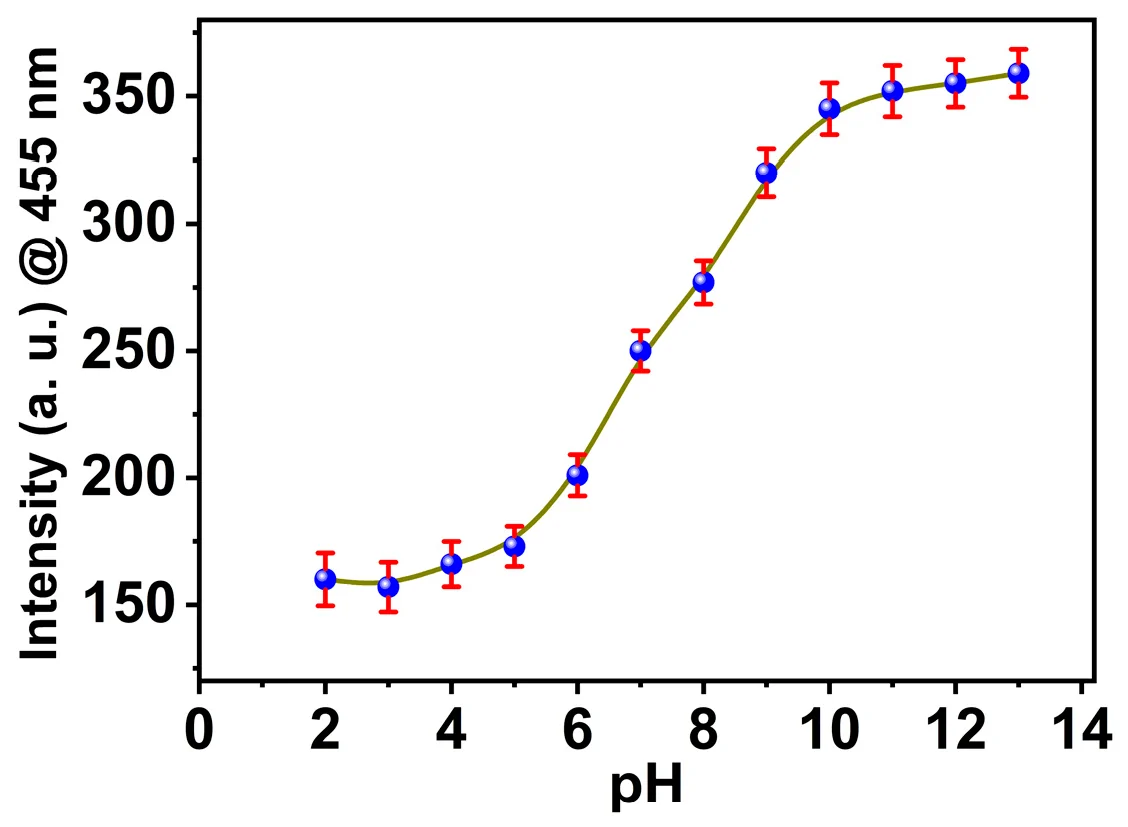
Effect of pH on the fluorescence intensity of probe 1 μM *AHQ* @455 nm under 364 nm excitation.

The effect of pH change on the ligand *AHQ* was tested in the pH range of 2–12. A weak emission for the ligand *AHQ* was observed in the pH range of 2–6 at 425 nm, and when the pH was increased by more than 6, the emission peak started to increase, with a small red-shift centred at 455 nm. In alkaline solution, the peak increased until it reached maximum emission at pH 12. Figure 2 presents the relationship between the fluorescence intensities of AHQ and the pH. The effect of pH on *AHQ* can be attributed to the existence of an excited-state intramolecular proton transfer mechanism (ESIPT). During the excitation process, photon-induced proton tautomerization occurs via internal proton transfer from the hydroxyl group to the oxygen atom of the carbonyl group to produce enol keto tautomerism as the oxygen atom is highly electronegative when withdrawn. As a result, an intramolecular hydrogen bond occurs during the excitation process. The large Stokes shift value of AHQ validates the ESIPT mechanism. The increase in fluorescence in the basic medium is due to intramolecular charge transfer (ICT) following the ESIPT process. In the basic medium, the AHQ molecule is deprotonated to form a phenoxide anion that is electron-rich and can act as a donor, while the benzene ring is electron-deficient and acts as the acceptor; the negative charge is distributed over the conjugated system in the ground state [42]. Resonance stabilization is enabled by extensive delocalization of the negative charge over the benzene p-electrons. At pH 11, the luminescence turns out to be a constant due to the complete deprotonation of the AHQ molecule.

### 3.4. Quantum Yield Measurements of AHQ

A molecule’s fluorescence quantum yield (QY) is a fundamental photophysical property that may be experimentally measured. The fluorescence yield is essential in several aspects of chemistry since it relies on the fundamental unimolecular rate constants for excited-state decay. The quantum yield (QY) was determined using the following equation, employing quinine sulfate as a reference in sulfuric acid (QY ≈ 55%) [43,44]. The quantum yield for *AHQ* was estimated as 0.19, with a limiting error for the quantum yield (±6%).

$$Q_{X}=Q_{R}\frac{A_{R}I_{x}n_{x}^{2}}{A_{X}I_{R}n_{R}^{2}}$$

where

• X refers to AHQ ligand;
• R refers to quinine sulfate as a reference;
• η refers to the refractive index at 25 °C;
• I refers to the surface area under the peak (integrated);
• A refers to absorbance peak maxima.

### 3.5. The Copper Sensing Approach

**Figure 3:**
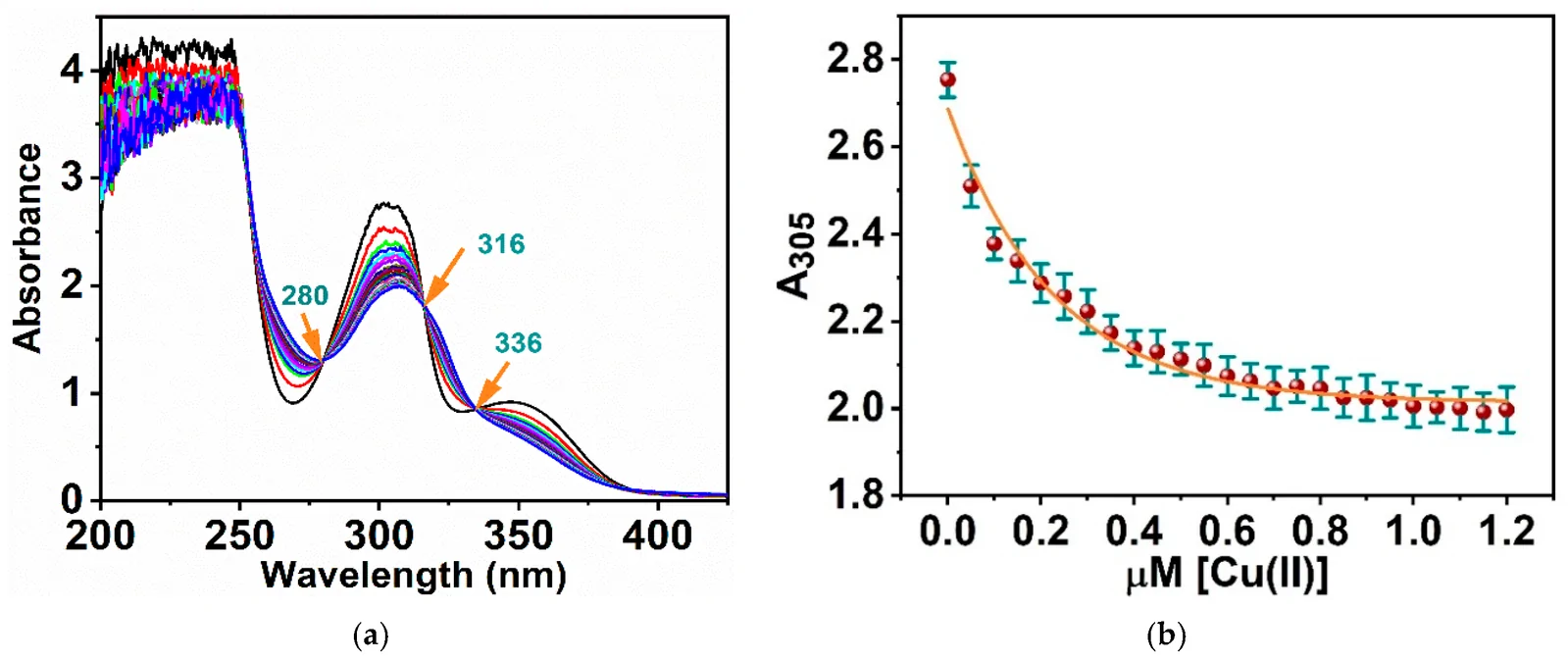
(**a**) UV-vis titration of probe *AHQ* (2 μM) in a solution with a pH of 7.4 with Cu(II) and (**b**) calibration curve of the absorbance of AHQ vs. [Cu(II)].

The *AHQ* luminescence reactivity to the Cu(II) ions was observed utilizing absorbance and fluorescence methods. The interactions between the Cu(II) ions and the *AHQ* molecules probe caused a considerable change in the absorbance peaks at 305 and 350 nm. In addition to these, two notable absorbance peaks can be attributed to π-π* and n-π* transitions [45]. Absorbances of 305 and 350 nm can be seen as the intensities of the two peaks decrease with the addition of copper ions (see Figure 3). The 305 nm absorption peak has a red-shift which is synchronized by the decrease in the peak. Additionally, the 350 absorption peak undergoes a blue-shift and disappears when Cu(II) ions are introduced to the *AHQ* probe. The two absorption peaks continue to exhibit synchronized behaviour due to the establishment of three isosbestic points at 280, 316, and 336 nm (Figure 3a). The chelation procedure involving metal ions and the AHQ active groups, including the sensitive chemical sensor’s oxygen and nitrogen atoms, is verified by the anomalous response of *AHQ* UV-vis spectra [46]. The change in the absorption of *AHQ* at 305 and 350 nm is significant to the concentration of Cu(II) over the dynamic range of 0.1 to 1.2 μM (Figure 3b). In particular, the absorption ratios remain constant at higher molar ratios of *AHQ* to Cu(II) (2:1 equivalent). As a result, the ratiometric detection method identifies a 2:1 stoichiometric ratio of *AHQ* in the Cu(II) interaction.

The apparently opposite directions of the calculated and observed spectral shifts are mutually consistent because they refer to two different electronic transitions. The higher-energy band near 350 nm of free AHQ has a substantial n-pi* character, arising from the non-bonding lone pairs of oxygen atoms. Upon chelation to Cu(II), these lone pairs are engaged in sigma-donation to the metal and are strongly stabilized (lowered in energy); this widens the n-pi* energy gap and therefore produces a hypsochromic (blue) shift and the eventual disappearance of the band, the same behaviour that n-pi* transitions display when the carbonyl/enol lone pair is hydrogen-bonded or a coordinated complex. The TDDFT excitations computed at 333–347 nm, by contrast, correspond to the newly created charge transfer (ICT/LMCT-type) transition of the complex, in which electron density is redistributed toward the copper centre; this transition is intrinsically weak (low oscillator strength) and, rather than generating a strong red-shifted absorption band, chiefly opens a non-radiative decay pathway that underlies the fluorescence turn-off. There is thus no contradiction: the strong ligand n-pi* band blue-shifts and vanishes as the oxygen lone pairs coordinate, while the weak metal-directed CT transition of the complex lies at lower energies but with negligible intensity. The intramolecular charge transfer character of AHQ itself is independently corroborated by its large Stokes shift (delta-lambda = 91 nm).

**Figure 4:**
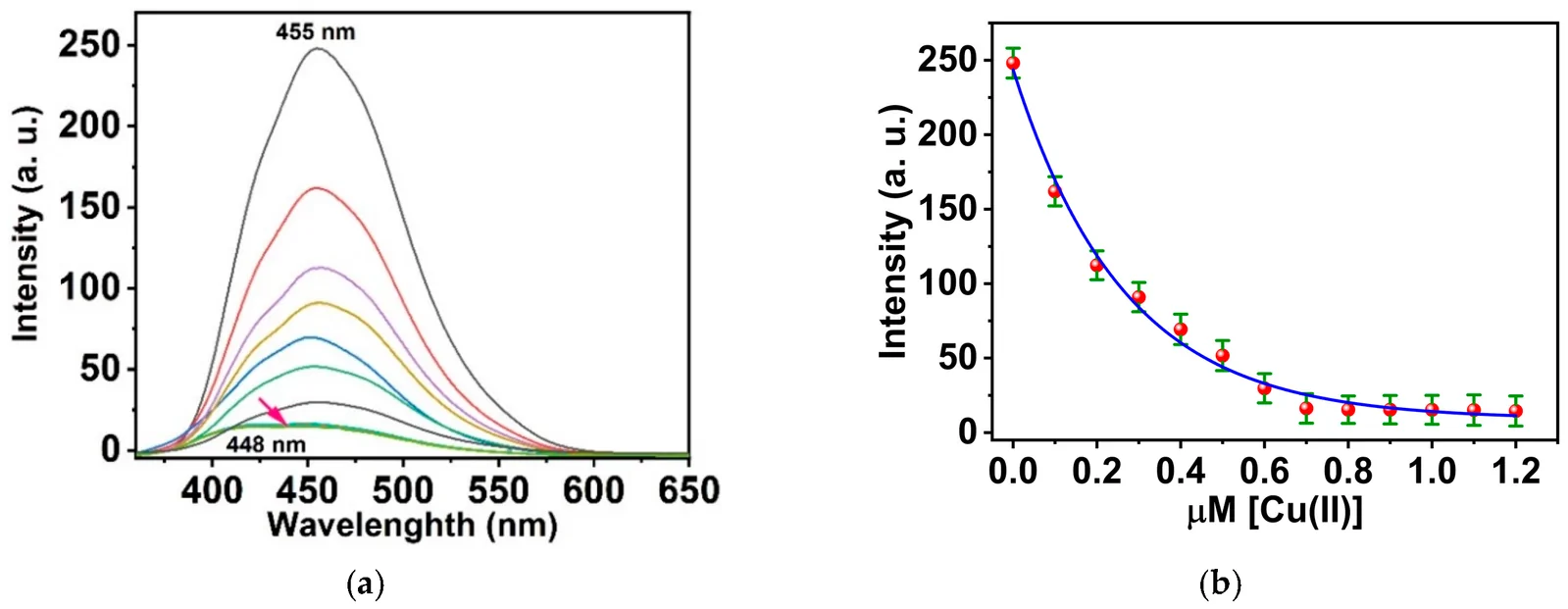
(**a**) Fluorescence titration of organic probe *AHQ* (2 μM) in a (40/60, *v*/*v*) DMSO-HEPES with a pH of 7.4 with Cu(II) and (**b**) caliberation curve of AHQ fluorescence intensity vs. [Cu(II)].

Moreover, the *AHQ* sensing molecule has luminous characteristics, with a prominent peak seen at 455 nm and an excitation wavelength (λ_exc_) of 364 nm. The optical properties of *AHQ* exhibit no overlap between excitation and luminescence, with a significant Stokes shift (Δλ = 91 nm) found. The significant Stokes shift diminishes self-quenching, which is essential for prospective application. The incremental adding of Cu(II):*AHQ* significantly diminished the fluorescence sensitivity of the conspicuous peak at 455 nm. Figure 4a illustrates that the magnitude of signal attenuation came to approximately 94.2% when 1 μM of Cu(II) was added. Furthermore, introducing Cu(II) ions to the *AHQ* sensing molecule within a range of 0 to 2 equivalents results in a significant quenching of the conspicuous emission peak, which shifts to 448 nm. Nonetheless, the fluorescence intensities stabilized when adding almost 2:1 ligand:Cu(II) ion equivalents. The luminescence titration exhibited a nonlinear correlation in the fluorescence emission titration curve, as shown in Figure 4b. Also, absorption titration findings indicated a 2:1 binding ratio among the *AHQ* sensing molecule and Cu(II) ions.

The observation of well-defined isosbestic points is fully compatible with the proposed 2:1 (AHQ:Cu(II)) stoichiometry. The formation of a bis-ligand complex proceeds stepwise, AHQ + Cu(II) <-> [Cu(AHQ)] followed by [Cu(AHQ)] + AHQ <-> [Cu(AHQ)2], and clean isosbestic points are retained whenever the mono-ligand intermediate does not accumulate to a spectroscopically significant concentration. Because the second binding step is favoured (positive cooperativity, K2 >= K1, as is common for chelating beta-ketoenolate ligands that complete the preferred coordination sphere of Cu(II)), the [Cu(AHQ)] intermediate is present only at trace steady-state levels, so the titration is dominated by the spectral interconversion of free AHQ and the final [Cu(AHQ)2] complex and behaves effectively as a two-state process. The 2:1 stoichiometry is not inferred from the isosbestic behaviour but is established independently by the continuous-variation (Job’s) plot and by the saturation of both the absorption and emission titrations at an AHQ:Cu(II) ratio of 2:1. The mono-ligand [Cu(AHQ)] and bis-ligand [Cu(AHQ)2] species share essentially the same chromophore since, in both, the AHQ ligand is perturbed in the same way by direct Cu-O coordination. They are therefore spectrally similar in absorption, and their sequential formation generates a single common set of isosbestic points rather than two distinct sets. This explains why a mono-ligand description reproduces the absorption behaviour of the 2:1 species, with the mono-ligand complex being the necessary precursor from which the bis-ligand complex is built.

### 3.6. Quenching Study

The Stern–Volmer equation was applied to study the fluorescence colliding quenching between Cu(II) and the AHQ chemical probe [47]:

*F*_0_/*F* = 1 + *K_sv_*[*Q*]

where

• F_0_ is the native *AHQ* luminescence;
• F is the *AHQ* luminescence in the presence of Cu(II);
• K_sv_ denotes the quenching constant;
• [Q] represents the Cu(II) concentration.

**Figure 5:**
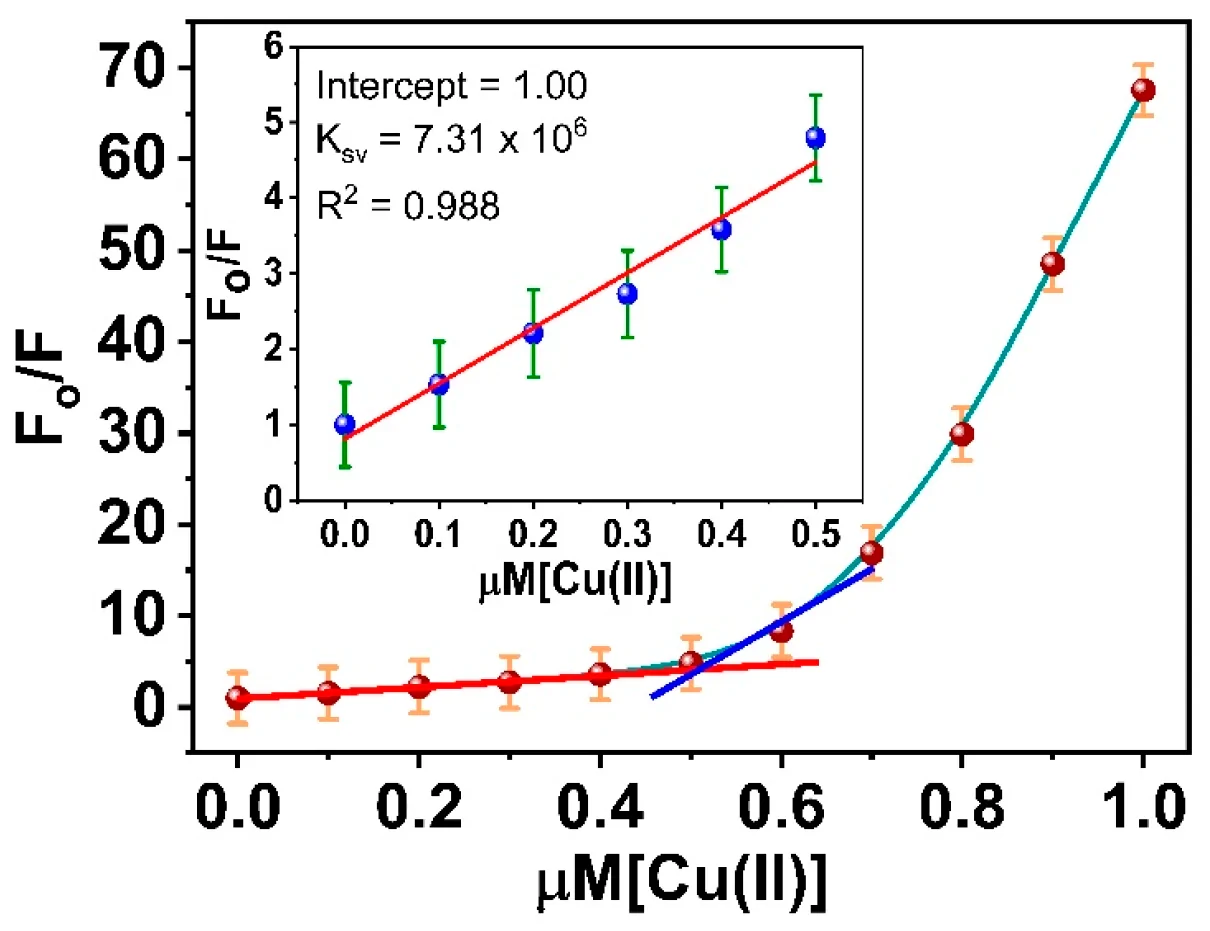
Stern–Volmer relationship for Cu(II)-induced quenching luminescence of the *AHQ*.

Data on quenching is presented as F_0_/F vs. [Q] (see Figure 5). A linear Stern–Volmer plot indicates the occurrence of collisional fluorescence quenching. Through a spacious range from 0 to 0.5 μM, R^2^ = 0.988% exhibited remarkable linearity, with a K_sv_ constant of 7.31 × 10^6^. The light quenching of *AHQ* by Cu(II) ions exhibits non-linearity beyond 0.5 μM. Such graphs are characteristic of scenarios where static quenching transpires. The Stern–Volmer relation exhibits linearity when static quenching serves as the only deactivation mechanism. Quenchers are present at concentrations ranging from 0.5 to 1 μM, contingent upon the kind of ion, and the quenching constants are characteristic of static quenching. Static quenching denotes the development of a nonfluorescent compound in the ground state between the *AHQ* and the Cu(II). Additionally, the two linear regions in Figure 5 are due to the 1:1 and 2:1 complexes. This complex exhibits temporal stability, distinguishing it from the non-complexed *AHQ* and the excited-state complex generated during static quenching.

### 3.7. Metal–Probe Binding Calculations

The Cu(II)–AHQ binding was investigated by employing the modified Stern–Volmer equation [15]:

F_0_/(F_0_ − F) = 1/A + 1/A∙K_b_

This was undertaken to examine the mechanism of chemical interaction between *AQH* and Cu(II), where

• F_0_ is native *AHQ* luminescence;
• F is *AHQ* luminescence in the presence of Cu(II);
• K_sv_ denotes the quenching constant;
• [Q] represents the Cu(II) concentration;
• *K_b_* is a binding constant [48];
• A is a numerical constant.

**Figure 6:**
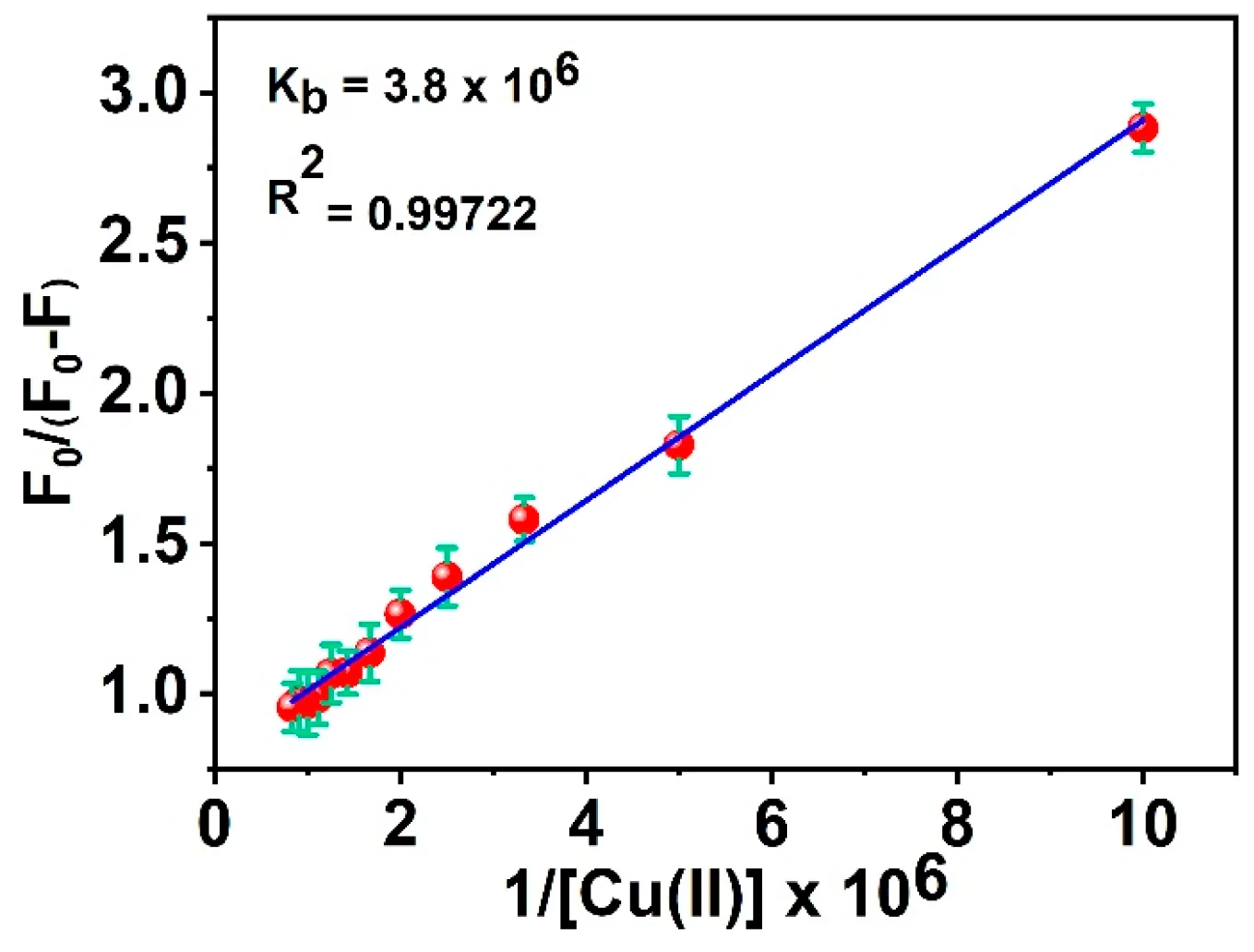
Modified Stern–Volmer equation for Cu(II)–AHQ interaction.

In a linear relationship (y = α + βx), y = F_0_/(F_0_ − F), α (intercept) = 1/A, β (slope) = 1/A, K_b_, x = 1/[Q]. K, estimated from α/β, was obtained when F_0_/(F_0_ − F) was plotted as a function of the 1/[Q] concentration. K_b_ was determined to be 3.8 × 10^6^ M^−1^ based on the fluorescence titration curves of the *AHQ* probe with Cu(II) ions (see Figure 6).

### 3.8. Validation Studies

**Figure 7:**
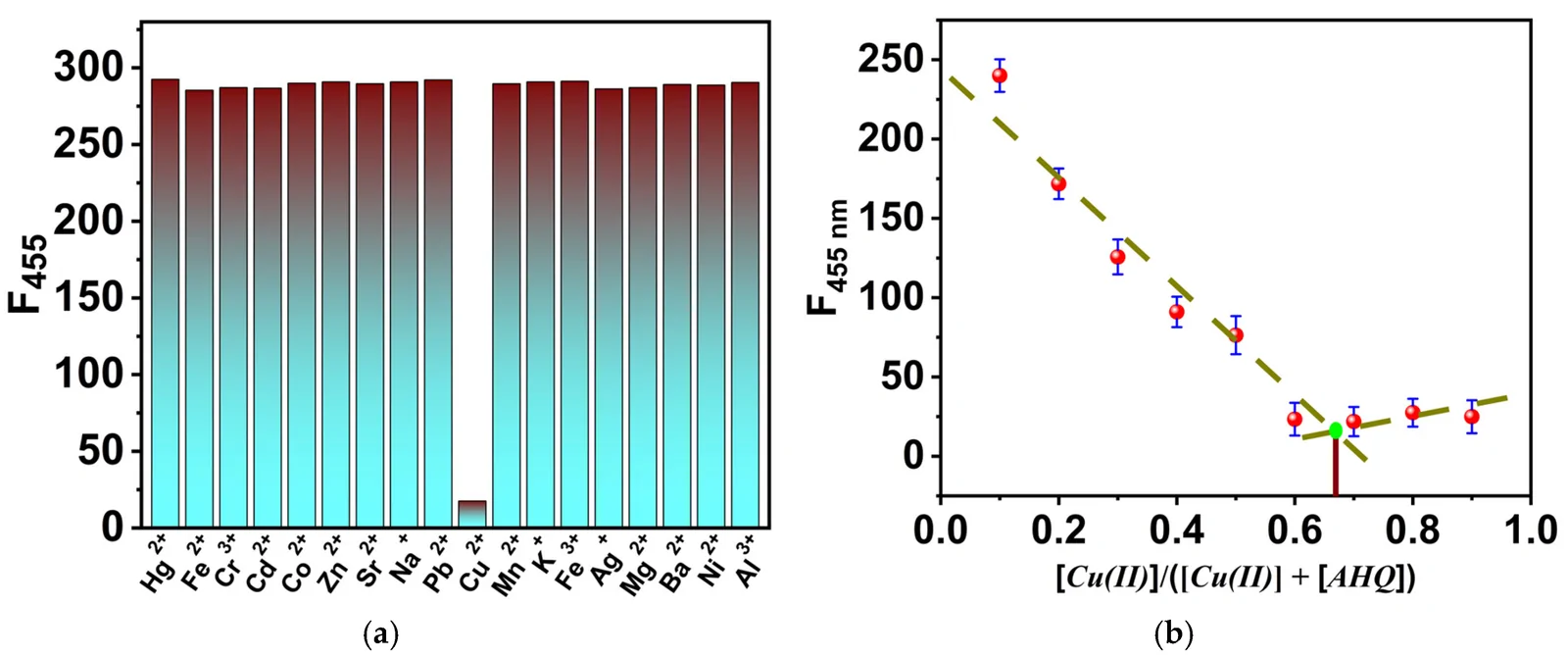
(**a**) Fluorescence response of *AHQ* to Cu(II) (1 μM) with other competitive metal ions (2 μM). (**b**) Job’s plot for determining the stoichiometry of the Cu(II)–*AHQ* complex using the metal molar ratio on the *x*-axis vs. the fluorescence intensity @455 nm on the *y*-axis.

To examine the interference capacity of several metal ions with respect to the reactivity of *AHQ* to Cu(II) metal ions, different alkali, alkaline earth, and transition metal ions were introduced to a reaction medium of *AHQ*–Cu(II) at a fixed ratio of 2:1 equivalent, and the *AHQ* emission was measured. Several metal ions were incorporated into a Cu(II):*AHQ* medium at a molar ratio of 1:2. The investigated metal ions show no substantial effect on the emission intensity of *AHQ*. Consequently, the reactivity of the examined metal ions does not disrupt the response of *AH*Q to Cu(II) ions using fluorescence intensity measurements in a (40/60, *v*/*v*) DMSO-HEPES with a pH of 7.4 (Figure 7a). Consequently, the *AHQ* sensing probe is an exceptionally sensitive and selective chemical sensor for detecting Cu(II) ions. The binding procedure and molar metal-to-ligand ratio were further analyzed using Job’s plot [49], based on the fluorescence values illustrated in Figure 7b. Job’s plot was constructed by varying the molar ratio of Cu (II) ions in the range of a 0:1 molar fraction.

### 3.9. Reversibility

Ethylaminetetraacetate (EDTA), a potent chelating agent for Cu(II) ions, was utilized to study the reversibility of the *AHQ* sensing probe. An equivalent volume of EDTA solution was gradually added to a solution containing a Cu-(*AHQ*) molecule with a molar ratio of 1:2 (metal to the ligand). This was done progressively. The titration and Job’s plot data indicate that the sensing species is a 1:2 Cu(II):AHQ complex. A plausible mechanism involves direct coordination of Cu(II) to the two carbonyl (oxo) sites of the AHQ ligand, with two AHQ molecules acting as bidentate donors arranged around a single Cu(II) centre. This Cu(II)-(AHQ)_2_ coordination environment promotes metal–ligand charge transfer (MLCT) and alters the conjugation and charge distribution within the quinolinone scaffold, leading to the characteristic changes in absorption and fluorescence response used for Cu(II) detection.

**Figure 8:**
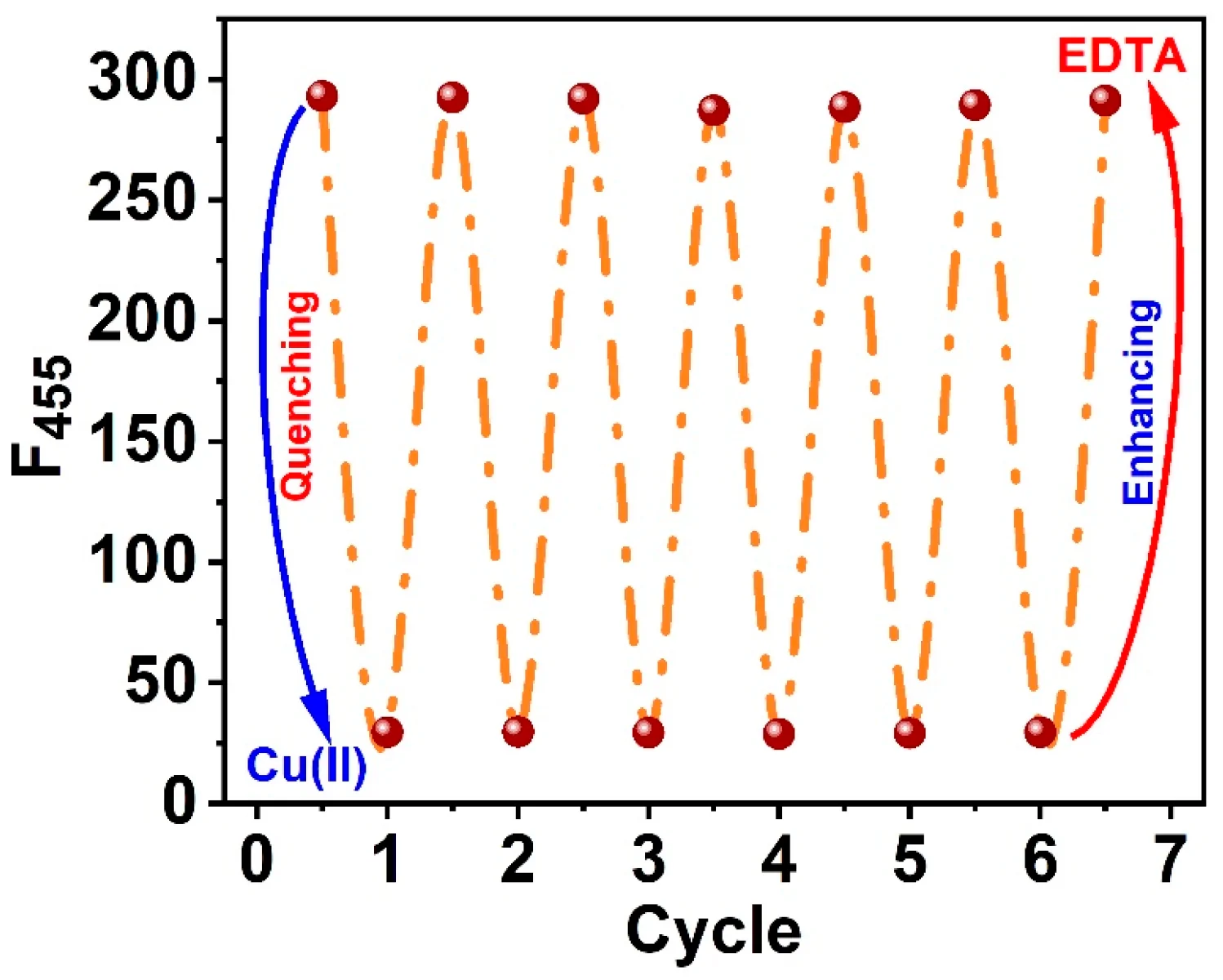
The introduction of EDTA restores the *AHQ* fluorescence.

As soon as EDTA was added, the molecules started exchanging *AHQ* molecules, immediately forming a Cu-EDTA complex (see Figure 8). Interestingly, the switch binding procedure was maintained by an increase in fluorescence brought about by the sensing probe *AHQ* that was released. Because of this, the emissions grew to around ≈97% of the native *AHQ* emissions.

### 3.10. Chemosensor Film

**Figure 9:**
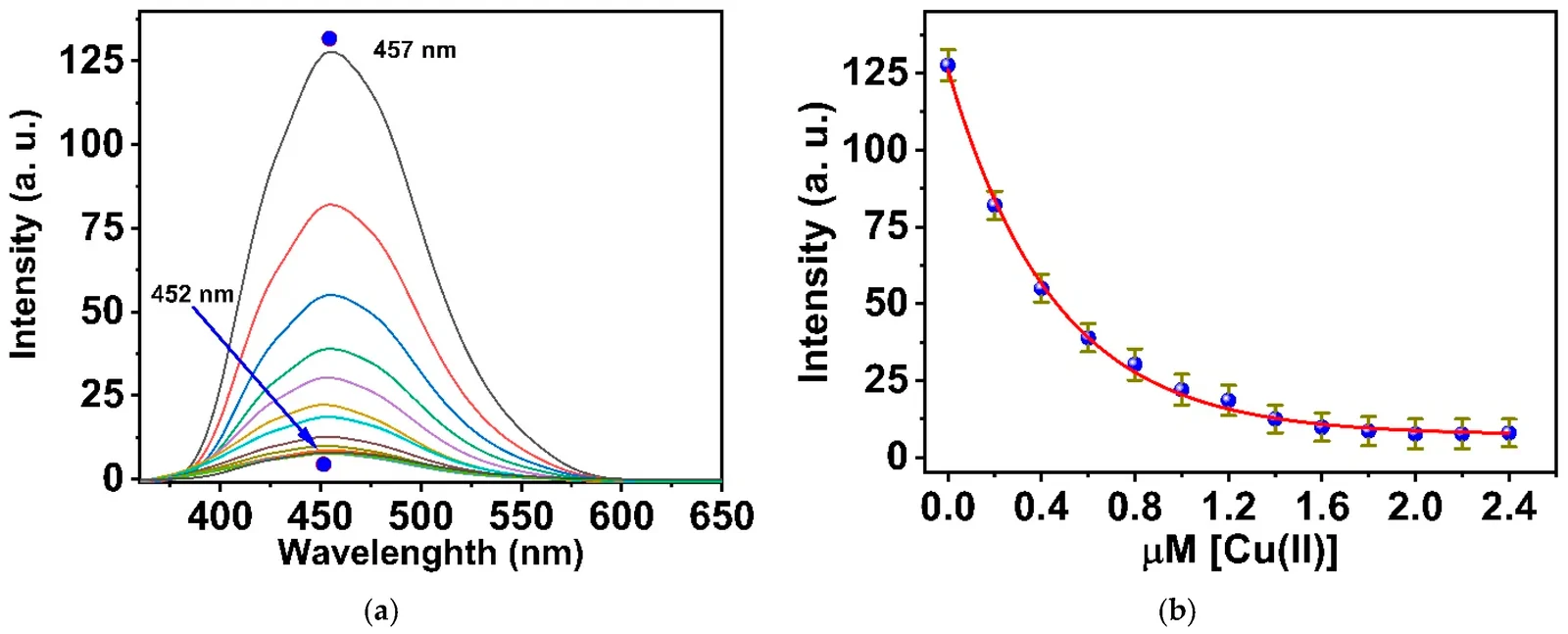
(**a**) Fluorescence titration of polymeric sensor film based on the organic probe *AHQ* with Cu(II) in 20 mM HEPES with a pH of 7.4 under 364 nm excitation. (**b**) Relationship between *AHQ* intensity and Cu(II) concentrations.

The luminescence characteristics of the chemosensor film have been measured in the presence of varying amounts of Cu(II) ions, as illustrated in Figure 9a. The sensor film exhibits emissions at 457 nm when immersed in a buffer solution with pH 7.4. Significant attenuation in the emission band at 457 nm was observed during fluorescence titration with the Cu(II) solution. The complex formation was confirmed by suppressing the fluorescence intensity of the principal emission peak at 457 nm. Additionally, the continuous addition of copper ions significantly quenched the emission intensity of the fluorescence band at 457 nm, as demonstrated in Figure 9b, which also demonstrates the correlation dependence of (I_457_) on [Cu(II)]. The quenching mechanism can be explained based on internal charge transfer (ICT), which is ascribed to the execution of complex procedures [50]. AHQ is a donor chelator that binds to Cu(II) as a receptor, the *AHQ* probe molecule exhibited exceptional attachment to Cu(II)within a concentration range of 0 to 2.4 μM. The LOD was approximated as 10.8 nM Cu(II) using the equation 3σ/k, where σ is the standard deviation of blank measurements, and k is the slope of the calibration curve [51,52]. Notably, this LOD is much lower than the Cu(II) concentration (31.5 μM) indicated in the WHO’s guidelines for drinking water characteristics [53]. Consequently, the optical chemosensor *AHQ* significantly impacts the detection of Cu(II).

The AHQ probe was examined as a sensor film for tracing Cu(II) metal ions in tap water samples. The analyzed water samples were directly used without any further treatment. Detection developed after samples were adjusted in 20 mM HEPES buffer solution with pH 7.4. Moreover, the genuine samples were spiked independently with some quantities of metal Cu(II). The collected results depend, memorably, on the standard curve from the sequence of experiments. The Cu(II) concentrations of the cassette water samples are determined. The data are shown in Table 1. The average detection recoveries ranged from 97.1% to 99.66% for Cu(II). The obtained results were compared with the ICP-MS data, proving that this approach for sensing Cu(II) in environmental samples is valuable.

**Table 1:** Real samples investigation based on *AHQ* sensor (*n* = 3).

Samples	Added Cu(II) µM	ICP-MS µM	Found µM	RSD (%)	Recovery (%)
Tap water	0.3	0.303	0.292	1.37	97.33
	0.9	0.904	0.897	1.11	99.66
	1.4	1.407	1.376	0.98	98.29
	2.0	2.015	1.942	0.91	97.10

### 3.11. Chemosensors Comparison with Recent Studies

To investigate the sensitivity and efficiency of the AHQ ligand towards Cu(II) ions, a comparison between the AHQ system and recently designed organic chromophores for Cu(II) ions was made in terms of the type of the binding with Cu(II) ions and the working medium. As shown in Table 2, the proposed system exhibits excellent performance compared with most of the currently developed optical probes for Cu(II).

**Table 2:** Comparison of recently designed chromophores for Cu(II) with the current work.

Sensor	Matrix/Medium	Detection Limit	Response Type	Ref.
L derived from vitamin B6 cofactor pyridoxal	DMSO:H_2_O (1:1, *v*/*v*)	1.89 μM	Fluorescence quenching (Cu(II))	[54]
rhodamine 6G acylhydrazine and 5-formyl-6-hydroxyl-4-methylcoumarin/2,4-dihydroxybenzaldehyde	EtOH/H_2_O (1:1)	0.592 and 0.977 μM	Fluorescence quenching	[55]
Dansyl derivative	DMSO:H_2_O (1:1, *v*/*v*).	0.979 μM	Fluorescence turn-on	[56]
Coumarin graphene probe	Aqueous solution	2 × 10^−15^ M	Fluorescence turn-on	[57]
Lawsone azo derivative	Aqueous solution	2.7 × 10^−7^ M	Fluorescence quenching	[58]
azo-phenol derivative probe	EtOH/H_2_O (*v*/*v*, 1:9)	7.2 × 10^−7^ M	Fluorescence turn-off	[59]
imidazoazine framework	MeOH/H_2_O (*v*/*v*, 2:8)	10^−7^ M	Fluorescence turn-off	[60]
tetraphenylethene (TPE) derivatives	DMSO:H_2_O, 1:99	2 × 10^−7^ M	Fluorescence quenching	[61]
9-(dicyanomethyl)anthracene	Aqueous	1.7 × 10^−9^ M	Fluorescence quenching	[62]
AHQ-based sensor (this work)	(40/60, *v*/*v*) DMSO-HEPES	5.6 nM	Fluorescence quenching (Cu(II))	This work

### 3.12. Theoretical Study

Theoretical computations were achieved by employing the Gaussian 09 programme package [63], utilizing the density functional theory (DFT) and time-dependent DFT (TDDFT) approaches to clarify the structural and electronic characteristics of the Cu^2+^–AHQ complex and their influence on the properties of fluorescence. The molecular geometries of AHQ and its copper-bound form (Cu–AHQ) were refined using the PBE1PBE functional, incorporating the 6-31G(d) basis set for non-metal elements and the LANL2DZ basis set for the copper atom. Vibrational frequency computations at the identical theoretical level verified the lack of imaginary frequencies, confirming the robustness of the refined geometries. To mimic the aqueous environment of experiments, TDDFT computations incorporated the SMD solvation model at the PBE1PBE/6-31+G(d)/LANL2DZ level.

**Figure 10:**
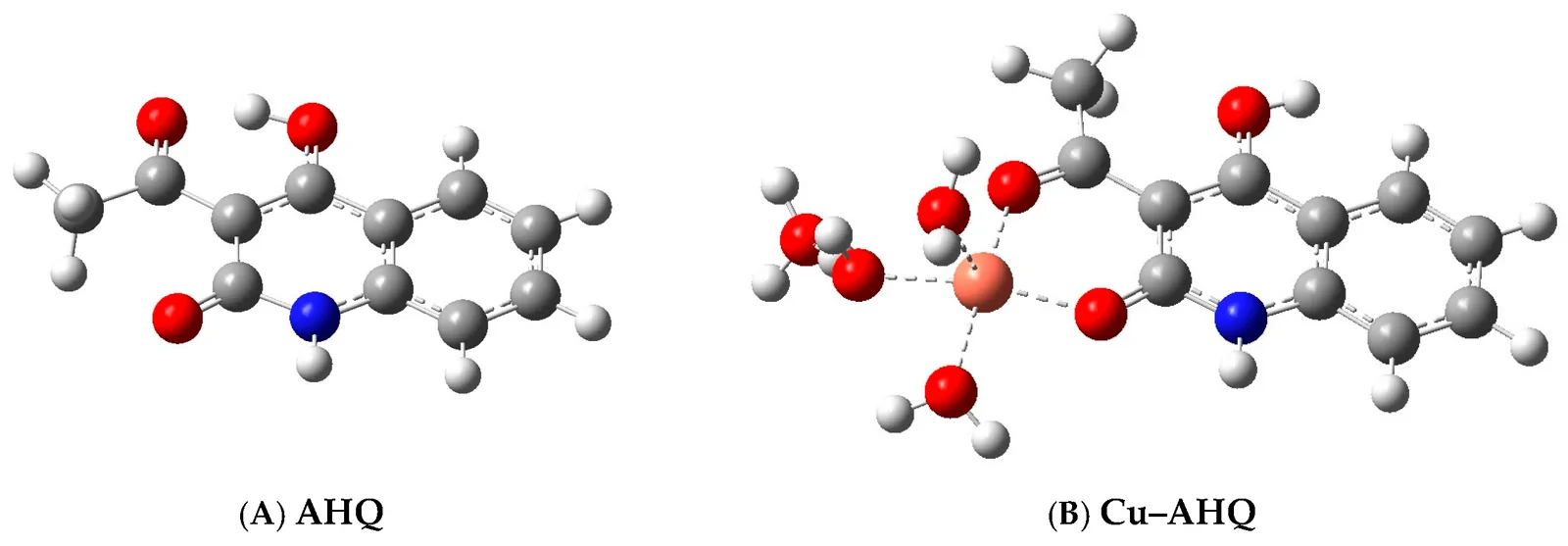
Model structures for (**A**) AHQ and (**B**) Cu–AHQ.

The refined geometry of the AHQ appears in Figure 10A, whereas the Cu–AHQ complex is illustrated in Figure 10B. Within this complex, the Cu^2+^ cation assumes a five-coordinate configuration, coordinating to AHQ through two oxygen atoms alongside three water ligands. The computed bond lengths include Cu–O (1.90 Å and 1.92 Å) and Cu–H_2_O (1.98 Å, 2.02 Å, and 2.26 Å). These distances underscore the efficient binding of Cu^2+^ to AHQ’s reactive sites.

The tautomer adopted in the present study, 3-acetyl-4-hydroxyquinolin-2(1H)-one, is the thermodynamically favoured form of these compounds, as documented for this system in [30]. Its preference arises because the 2-oxo group is a cyclic amide (lactam) that is strongly stabilized by amide (N-C=O) resonance with the nitrogen ring, in the same way that the lactam form dominates over the hydroxy (iminol) form in 2-pyridone and in 2-quinolones generally. This stabilization is reinforced by a planar, six-membered, intramolecularly hydrogen-bonded beta-ketoenol ring formed between the 4-hydroxy group and the adjacent 3-acetyl carbonyl, so the ligand strongly resists tautomerization to the 2-hydroxy-4-oxo form.

**Figure 11:**
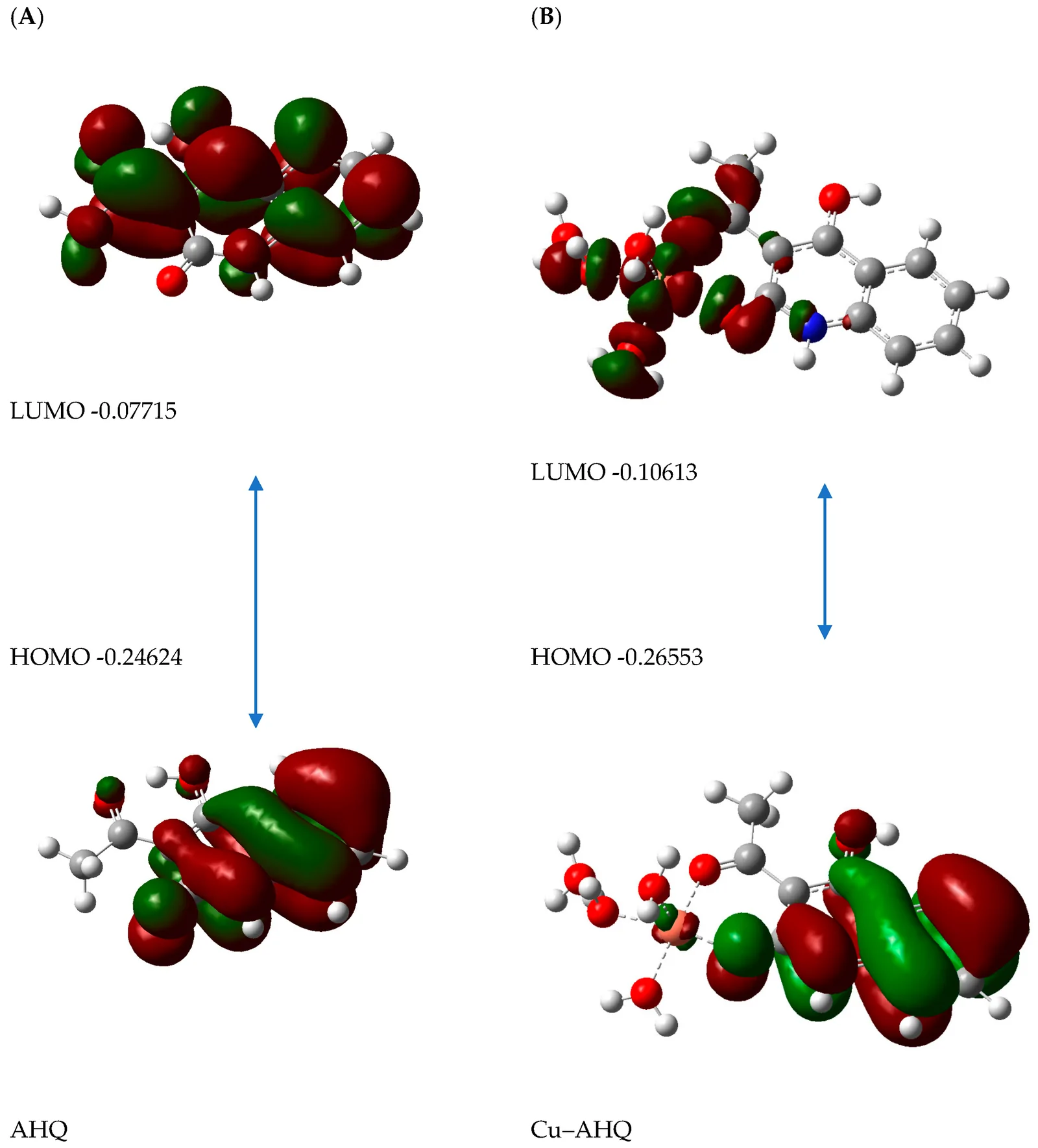
Frontier orbitals and their energies for (**A**) AHQ and (**B**) Cu–AHQ.

TDDFT analyses offered details on the electronic excitations in AHQ and Cu–AHQ, with the frontier molecular orbitals (FMOs) displayed in Figure 11. The essential excitation details, such as wavelengths and oscillator strengths, are outlined in Table 3. In AHQ, the HOMO and LUMO electron densities are mainly situated within the aromatic frameworks. Conversely, the Cu–AHQ complex demonstrates intramolecular charge transfer (ICT), featuring HOMO density primarily within the fused aromatic system, with LUMO density spread over the copper site and attached ligand atoms. The diminished oscillator strength observed in the Cu–AHQ complex (Table 3) aligns with fluorescence quenching, attributable to Cu^2+^ binding, which modifies the electronic configuration and inhibits emissive processes.

The mono-ligand [Cu(AHQ)(H_2_O)_3_] structure was adopted as a computationally tractable, minimal representation of the copper–AHQ chromophore. Because the two AHQ ligands in [Cu(AHQ)_2_] bind through the same O,O′-chelate pocket and are electronically equivalent, the essential perturbation of the ligand frontier orbitals, and hence the origin of the quenching, is already captured by a single Cu–AHQ unit, with the aqua ligands acting as surrogates for the remaining donor atoms of the coordination sphere; adding the second, electronically equivalent AHQ does not qualitatively change the frontier-orbital picture. The excited-state (emission) TDDFT of the complex was not pursued because the Cu(II)-bound form is essentially non-emissive; the open-shell, paramagnetic d9 Cu(II) centre introduces low-lying, metal-centred (d-d), and charge transfer states that provide efficient non-radiative deactivation channels, so the complex does not possess a populated, radiative excited-state whose geometry could be meaningfully optimized. The ground-state absorption TDDFT is therefore sufficient to rationalize the observed turn-off response, which reflects suppression of the ligand emission upon coordination rather than the appearance of a new emissive band.

**Table 3:** TDDFT outcomes of certain transitions, including associated absorbance energies and oscillator strengths.

Complex	Transition	λ (nm)	Oscillator Strength
AHQ	HOMO > LUMO	333	0.0999
Cu–AHQ	HOMO(b) > LUMO(b)	347	0.0011

## 4. Conclusions

In conclusion, a new type of chemosensor based on 3-acetyl-4-hydroxy-2-quinolone was synthesized and characterized for the recognition of Cu ions. It was found that *AHQ* showed a remarkable selectivity and sensitivity response towards Cu(II) ions in a (40/60, v/v) DMSO-HEPES with a pH of 7.4 using fluorescence and UV-vis methods. Furthermore, the present sensor determined the Cu(II) concentration of various water samples. The proposed chemosensor offers a novel method for developing a chemical probe with substantial luminescence changes. This is evidenced by the significant Stokes shift of 91 nm observed in the colourimetric and absorption *AHQ* sensors. The *AHQ* can detect Cu(II) ions with exceptional sensitivity, high selectivity, and a detection limit (LOD) of 10.8 nM. Copper ions and *AHQ* in nano-concentrations exhibited an exceptional linear relationship that could be employed to measure Cu(II) ions quantitatively. *AHQ* chelates Cu(II) ions in a 1:2 metal–ligand ratio, as demonstrated by Job’s experiments. The optical chemosensor’s mechanism depends on the chelation of Cu(II) by the *AHQ* chemical probe and the production of the *AHQ*–Cu(II) complex. DFT and TDDFT prove that the quenching of *AHQ* luminescence in the presence of Cu(II) ions occurs through ICT throughout the *AHQ* due to the binding metal ions. This chemical sensor, which is based on an *AHQ* sensor, has the potential to offer a distinctive approach to the detection of Cu(II) in the environment for research purposes.

## Data Availability

All data will be made available upon reasonable request.

## Supplementary Materials

The following supporting information can be downloaded at https://www.mdpi.com/article/10.3390/bios16080436/s1: Figure S1: H-NMR analysis of AHQ, Figure S2: 13C analysis of AHQ, and Figure S3: MS analysis of AHQ.
