# Supplementary material for: Novel Optical Sensor-Based Organic Chromophore for the Detection of Copper Ions
Source: Biosensors (Basel). 2026 Aug 12;16(8):436. doi: 10.3390/bios16080436 (PMC13511574; doi:10.3390/bios16080436)
Supplement: Supplementary file 1 [file biosensors-16-00436-s001.zip › biosensors-4397197-supplementary.pdf]

Supporting Information

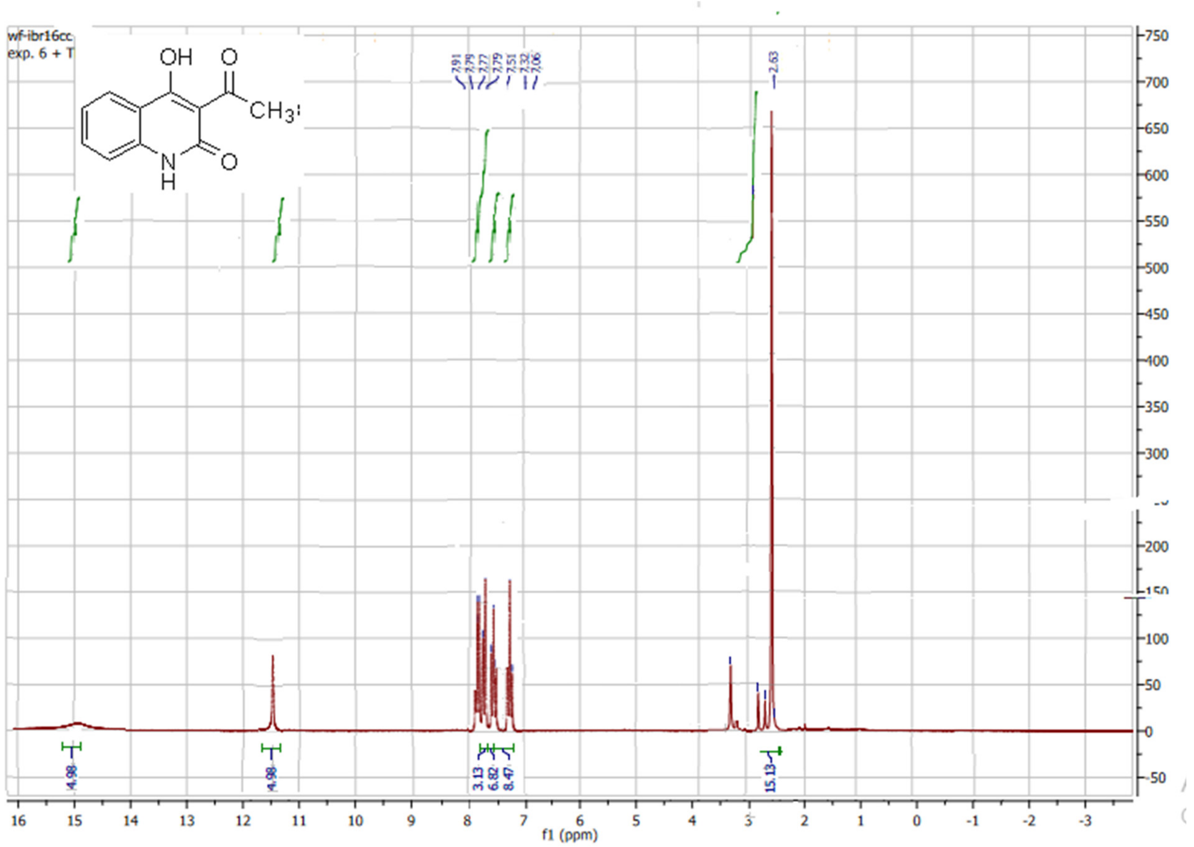

Figure S1. H-NMR analysis of *AHQ*

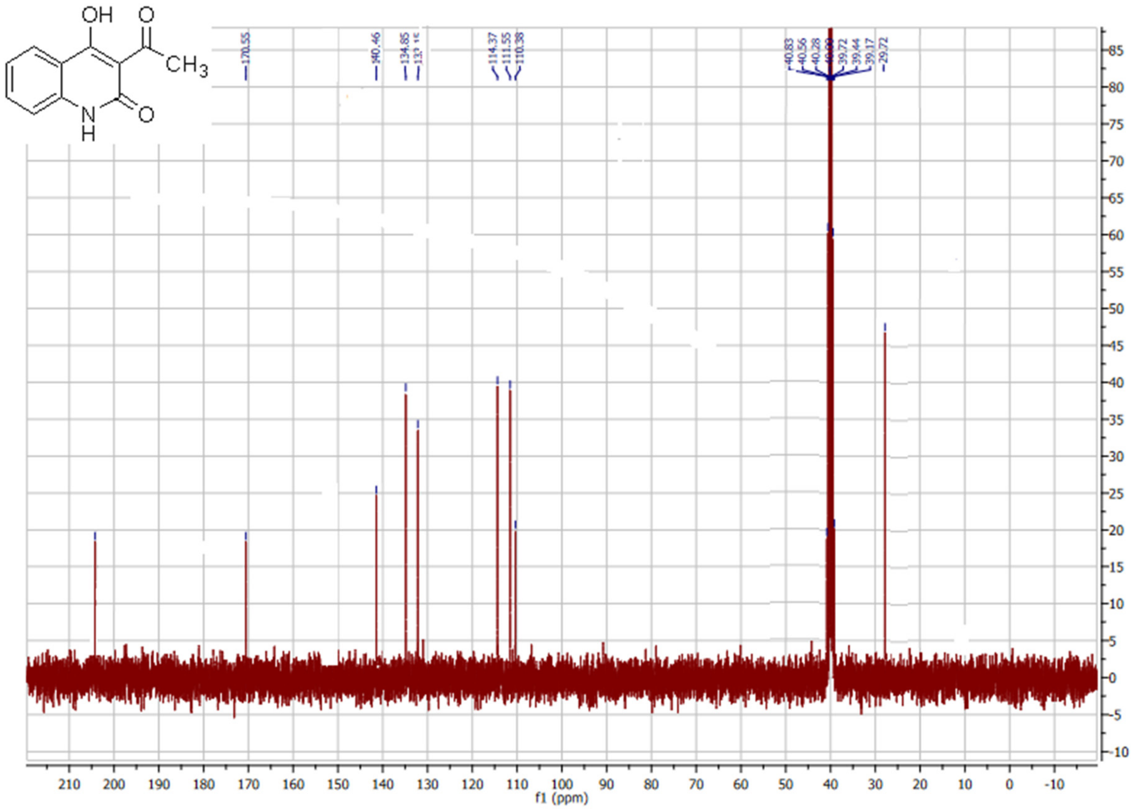

Figure S2. ^{13}C analysis of *AHQ*

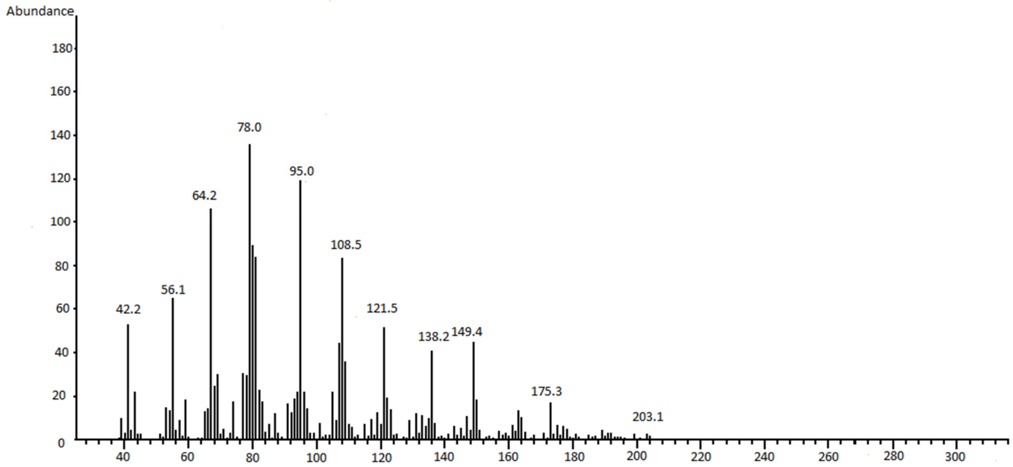

Figure S3. MS analysis of *AHQ*
